# Improving the Dissolution Rate and Bioavailability of Curcumin via Co-Crystallization

**DOI:** 10.3390/ph17040489

**Published:** 2024-04-11

**Authors:** Hao Wang, Chenxuan Zheng, Fanyu Tian, Ziyao Xiao, Zhixiong Sun, Liye Lu, Wenjuan Dai, Qi Zhang, Xuefeng Mei

**Affiliations:** 1 School of Chinese Materia Medica, Nanjing University of Chinese Medicine, 138 Xianlin Avenue, Nanjing 210023, China; 20211107@njucm.edu.cn (H.W.); 20211120@njucm.edu.cn (F.T.); zyxiao@njucm.edu.cn (Z.X.); 2 Pharmaceutical Analytical & Solid-State Chemistry Research Center, Shanghai Institute of Materia Medica, Chinese Academy of Sciences, Shanghai 201203, China; 406700210021@email.ncu.edu.cn (C.Z.); 406700220010@email.ncu.edu.cn (Z.S.); luliye@simm.ac.cn (L.L.); daiwenjuan@simm.ac.cn (W.D.); 3 School of Pharmacy, Jiangxi Medical College, Nanchang University, Nanchang 330006, China

**Keywords:** co-crystal, curcumin, L-carnitine, dissolution, bioavailability

## Abstract

Curcumin (CUR) is a natural polyphenolic compound with various pharmacological activities. Low water solubility and bioavailability limit its clinical application. In this work, to improve the bioavailability of CUR, we prepared a new co-crystal of curcumin and L-carnitine (CUR-L-CN) via liquid-assisted grinding. Both CUR and L-CN have high safe dosages and have a wide range of applications in liver protection and animal nutrition. The co-crystal was fully characterized and the crystal structure was disclosed. Dissolution experiments were conducted in simulated gastric fluids (SGF) and simulated intestinal fluids (SIF). CUR-L-CN exhibited significantly faster dissolution rates than those of pure CUR. Hirshfeld surface analysis and wettability testing indicate that CUR-L-CN has a higher affinity for water and thus exhibits faster dissolution rates. Pharmacokinetic studies were performed in rats and the results showed that compared to pure CUR, CUR-L-CN exhibited 6.3-times-higher AUC_0–t_ and 10.7-times-higher C_max_.

## 1. Introduction

**Figure 1:**
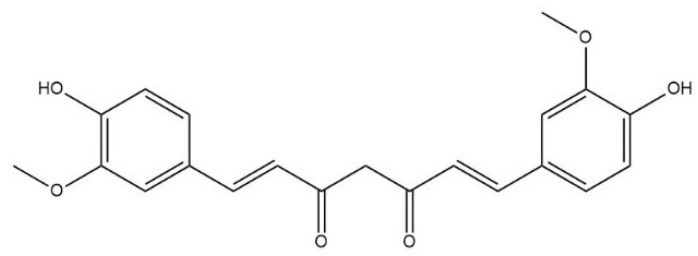
Chemical structure of CUR.

Curcumin (CUR) is a natural phenolic antioxidant extracted from the rhizome of ginger plant, turmeric, zedoary, and mustard [1]. It is a diketone compound, it has an unsaturated aliphatic chain and two aromatic rings (Figure 1), and is commonly used as a seasoning, an edible coloring, and medicine [2]. CUR is a biopharmaceutics classification system (BCS) class 4 compound with low water solubility and poor permeability. CUR has several potential drug applications, such as liver protection [3,4] and protection against diabetes [5,6], obesity [7,8,9], infertility [10], and depression [11,12]. Among them, liver protection is the most famous treatment effect. Sharifi et al. [13] reported a double-blind, placebo-control, single-center trial. The results showed that non-alcoholic fatty liver disease (NAFLD) patients with moderate-to-high hepatic steatosis oral CUR had significantly reduced blood concentrations of triglyceride (TG), total cholesterol (TC), low-density lipoprotein cholesterol (LDL-C), aspartate aminotransferase (AST), and alanine aminotransferase (ALT). Another set of clinical trials performed by Panahi et al. [14] also presented a similar clinical effect of CUR on the NAFLD patients. These clinical trial results confirmed the liver protection of CUR. CUR also presents excellent safety. In the USA, it is certified as “generally recognized as safe” (GRAS) [15]. It has been shown in three different phase I clinical trials that CUR can be well tolerated when taken in doses of up to 12 g per day [16]. 

CUR has excellent clinical effects and safety; however, its low bioavailability limits its application and development. Low absorption of CUR has been reported in several studies [17,18,19]. In 2007, Yang et al. [20] studied the pharmacokinetics of CUR in rats using both intravenous administration and oral administration, and the oral bioavailability of CUR was calculated as about 1%. Several factors may result in the low bioavailability of CUR, such as low water solubility, poor permeability, high metabolic rate, and rapid elimination [21,22]. Numerous techniques have been used to improve the bioavailability of CUR. Solubility enhancement is the most common method, and techniques for improving dissolution properties, such as nanoparticles [23], phospholipid complexes [24], solid dispersions [25], liposomes [26], and adjuvants [27], have been widely reported. Increasing permeability is another way to improve bioavailability. Cui et al. [28] prepared curcumin-loaded microemulsion. Compared with CUR, the microemulsion has a 2.5-times-higher absorption permeability. Piperine was added to mitigate the effects of metabolism on CUR absorption. Wang et al. [29] compared the bioavailability of CUR and CUR with piperine. The results showed that piperine could significantly improve the absorption of CUR. To slow down elimination of CUR, Najeh et al. [30] synthesized nanoparticles with polylactic-co-glycolic acid and polyethylene glycol. The nanoparticles significantly extended the mean half-life of CUR by four to six hours. However, nanoparticles and solid dispersions require a large number of carrier excipients, liposomes have stability problems in biological systems, and the preparation of phospholipid complexes requires the complexation of the drug with phospholipids at a certain temperature and the removal of solvents, which may cause degradation of CUR. Therefore, solving the problem of poor absorption of CUR is still a challenge.

Recently, co-crystallization has been labeled as a promising new method with which to enhance the solubility of CUR. Several co-crystals of CUR were reported, such as CUR-resveratrol co-crystal [31], CUR-ascorbic acid co-crystal [32], CUR-trimesic acid co-crystal [33], CUR-phloroglucinol co-crystal [34], and CUR-4,4′-bipyridine co-crystal [35]. However, there are no reports on the bioavailability of these co-crystals. Guangying L et al. [36] prepared a series mixture comprising curcuminoid or turmeric (curcuma longa) extract and L-carnitine (L-CN) with improved bioavailability to prevent or eliminate hangovers. L-CN is an endogenous molecule that promotes the conversion of fat into energy and plays an important role in the oxidative breakdown of fat, weight loss, and anti-fatigue [37]. The U.S. Expert Review Board has rated L-CN as safe as a nutritional supplement for adults at a dose of 20 mg/kg/day. Both CUR and L-CN have high safe dosages and have a wide range of applications in liver protection [38,39] and animal nutrition [40,41]. In this work, we prepared a new co-crystal of CUR with L-CN (CUR-L-CN) to improve its bioavailability. The crystal structure of the co-crystal was analyzed in detail via single crystal X-ray diffraction (SCXRD), and the co-crystal was thoroughly characterized via powder X-ray diffraction (PXRD), Fourier transform infrared (FTIR) spectroscopy, differential scanning calorimetry (DSC), thermogravimetric analysis (TGA), and dynamic vapor sorption (DVS). The dissolution properties and bioavailability of CUR and the co-crystal were also compared.

## 2. Results

### 2.1. PXRD Analysis and Confirmation of Co-Crystal Formation

**Figure 2:**
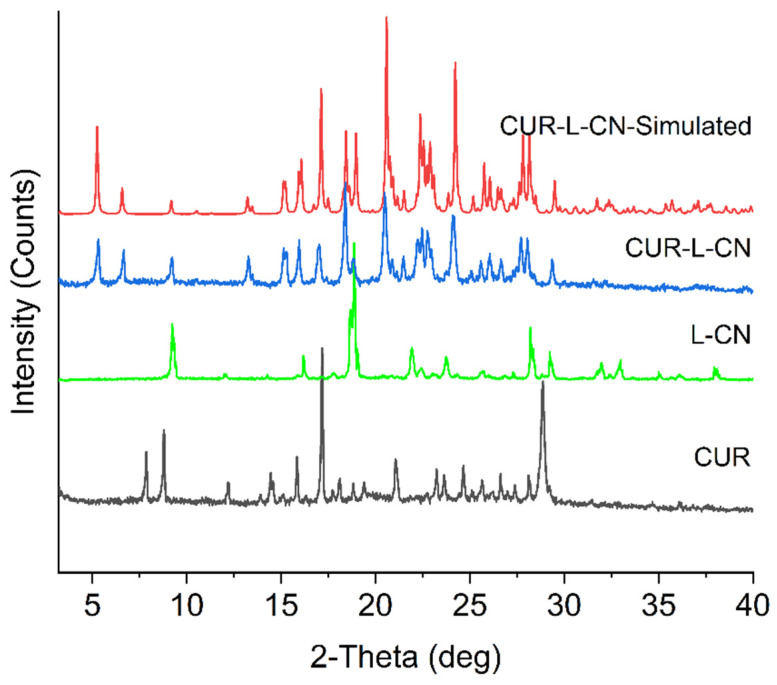
Compared PXRD patterns for CUR and its co-crystal.

**Figure 3:**
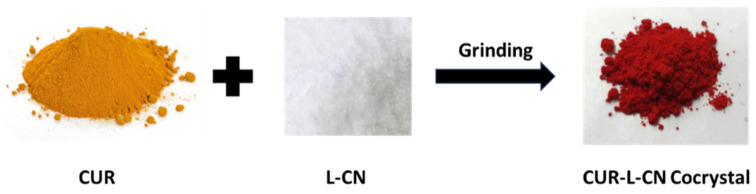
Color change of CUR in the co-crystallization process.

CUR is a polymorphic compound, and three polymorphs of CUR have been reported [42]. Among them, form I is the most stable form, and it is the marketed form. Forms II and III are unstable; they are easily changed to form I during storage. In this work, the co-crystal was prepared with CUR form I and L-CN via grinding. The synthesis was repeated more than ten times and the materials obtained all have identical properties. CUR, L-CN, and CUR-L-CN co-crystal were checked by PXRD, and the co-crystal formation could be confirmed by comparing their diffraction patterns. The PXRD patterns of CUR form I, L-CN, and CUR-L-CN co-crystal are shown in Figure 2. The results show that the co-crystal exhibited significantly different PXRD profiles compared to the CUR and its corresponding ligand L-CN, indicating the creation of a new phase. In addition, the experimental PXRD pattern of the CUR-L-CN co-crystal powder is in good agreement with the simulated pattern calculated from crystallographic data, confirming the generation of a high-purity phase. The co-crystal formation can also be confirmed by the color change during the co-crystallization process (Figure 3). Before co-crystallization, the CUR is a yellow powder, and the L-CN is a white crystal. However, after being mixed and ground, the color turns to red. This color change also confirms the formation of the co-crystal. 

### 2.2. Crystal Structure Analysis

**Figure 4:**
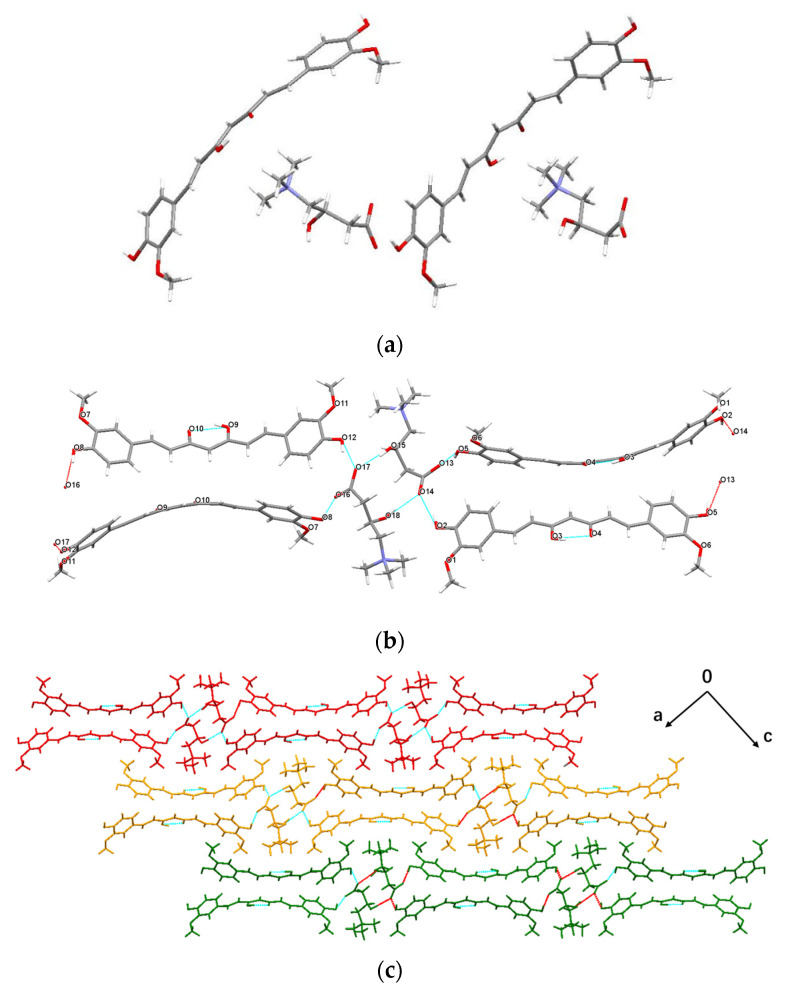
Crystal structure of CUR-L-CN co-crystal: (**a**) asymmetric unit; (**b**) hydrogen bonding interaction; (**c**) packing arrangement viewed along the b-axis.

Crystal structures of three CUR polymorphs have been reported [42]. Polymorphs I–III are crystallized in the monoclinic space group P2/n, the orthorhombic space group Pca2_1_, and the orthorhombic space group Pbca, respectively. They present a similar hydrogen bonding method, and the CUR molecules in the crystals are connected via O–H···O and C–H···O interactions. In comparation, CUR-L-CN presents a significantly different crystal structure. Crystallographic data and refinement details for CUR-L-CN co-crystal are listed in Table 1. It is solved in the monoclinic P2_1_ space group, and the asymmetric unit contains two CUR molecules and two L-CN inner salts (Figure 4a). In the crystal, two L-CN molecules are combined by hydrogen bonds O_15_–H···O_17_ (d = 2.652 Å) and O_18_–H···O_14_ (d = 2.743 Å) to form a dimer (Figure 4b). The CUR molecules are linked by the L-CN dimers with hydrogen bonds O_2_–H···O_14_ (d = 2.577 Å), O_5_–H···O_13_ (d = 2.534 Å), O_8_–H···O_16_ (d = 2.532 Å), and O_12_–H···O_17_ (d = 2.546 Å) to form infinite one-dimensional chains. These one-dimensional chains arrange along the c-axis to build the two-dimensional structure of the co-crystal (Figure 4c). In addition, there are also intramolecular hydrogen bonds O_3_–H···O_4_ (d = 2.482 Å) and O_9_–H···O_10_ (d = 2.492 Å) in the CUR molecules.

### 2.3. Thermal Analysis

**Figure 5:**
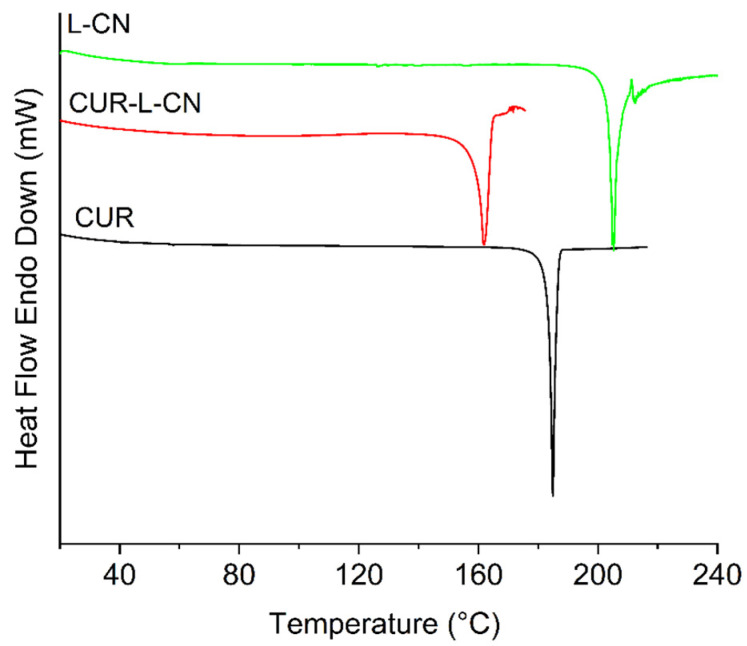
DSC curves of CUR, L-CN and CUR-L-CN.

Thermal analysis is used to examine the formation of new phases because the thermal behavior of the newly formed material shows different characteristics compared to the primary material. The overlaid DSC curves of the CUR, CUR-L-CN co-crystal, and ligand L-CN are presented in Figure 5. CUR has a melting point of 184.6 °C, and L-CN exhibited a melting point at 204.8 °C. In comparation, the melting point of the co-crystal is at 161.8 °C, which is far below the melting point of the two raw materials. The lower melting point indicates a lower lattice energy, and this may be the reason that the co-crystal dissolves much faster. TGA plots of the CUR-L-CN co-crystal and its corresponding ligands are provided in Figure S1. The TGA diagram of the co-crystal does not present any mass loss before its melting, indicating that it is a non-solvated form. 

### 2.4. FTIR Spectroscopy

**Figure 6:**
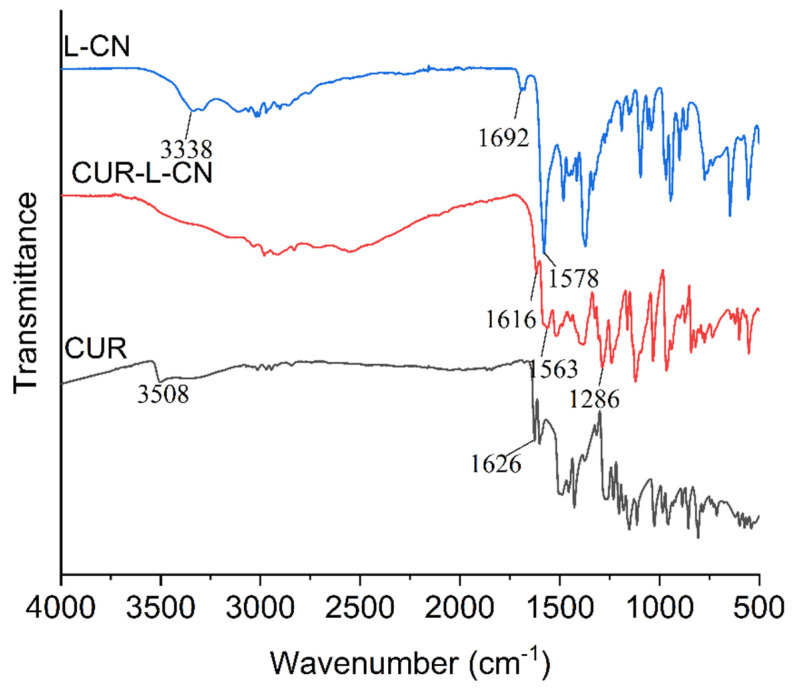
FTIR spectra of L-CN, CUR, and CUR-L-CN.

The overlaid FTIR spectra of CUR, L-CN, and CUR-L-CN are shown in Figure 6. The FTIR spectra of CUR display characteristic signals at 3508 cm^−1^, 1626 cm^−1^, 1601 cm^−1^, and 1426 cm^−1^, attributable to phenolic O–H stretching frequencies, C=O stretching, C=C aromatic groups stretching, and in-plane bending of the enol C–O, respectively. Compared with CUR and L-CN, the co-crystal presents a significantly different FTIR spectrum. The peaks at 3300 cm^−1^–3600 cm^−1^, corresponding to the O–H stretching vibration, have disappeared. The peak representing C=O stretching of L-CN carboxylate groups is red-shifted from 1578 cm^−1^ to 1563 cm^−1^. The peak corresponding to the C=O stretching of CUR carbonyl groups is red-shifted from 1626 cm^−1^ to 1616 cm^−1^. These red shifts indicate the strong hydrogen bond interaction between CUR and L-CN.

### 2.5. Hygroscopicity and Stability

**Figure 7:**
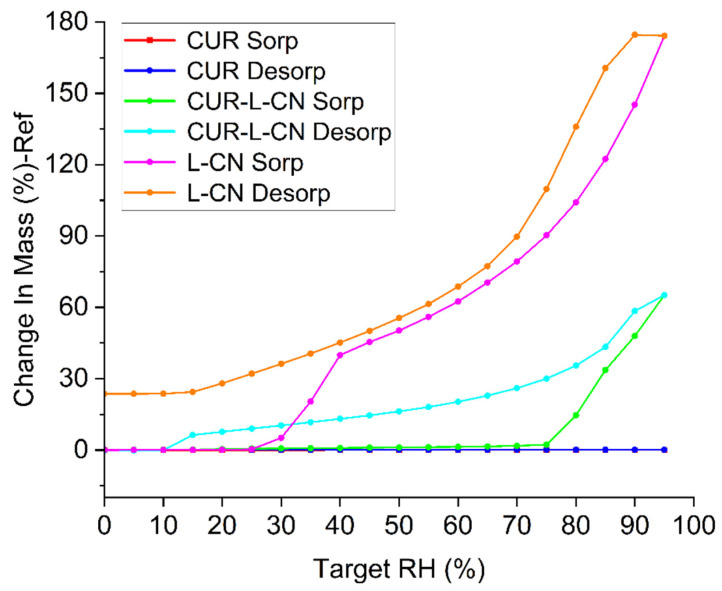
DVS isotherms of different powders at 25 °C.

The hygroscopicity of the drug is essential to ensure adequate stability in the solid form. Crystal engineering plays an increasingly important role in improving the hygroscopicity of drugs. L-CN is a serious moisturizing substance and high hygroscopicity is one of its well-known drawbacks. The DVS isotherms for CUR, L-CN, and CUR-L-CN are illustrated in Figure 7. It is shown that there is nearly no mass change of CUR during 0%-95% RH, indicating its non-hygroscopic nature. Consistent with previous reports, L-CN presents obviously high hygroscopicity. Above 30% RH, the moisture attraction of L-CN increases rapidly. At 40% relative humidity (RH), the water uptake of L-CN is 39.9%; at 95% RH, the water content rapidly increases to 174.2%. When the RH decreases to 0%, there is more than 20% (23.6%) water that cannot be removed. In comparation, the co-crystal exhibits much better hygroscopicity. Below 75% RH, CUR-L-CN presents very little water absorption. At 40% RH, the water uptake of CUR-L-CN is 0.87%. At 75% RH, the water content of CUR-L-CN is 2.26%. However, above 75% RH, the water absorption of CUR-L-CN increases rapidly. At 95% RH, the water uptake of CUR-L-CN reaches 65.2%. The inflection point at 75% RH indicates that there may be a phase transformation. After the DVS experiments, the residual sample of CUR-L-CN was checked via PXRD (Figure S2). The results show that the co-crystal was converted to CUR form I. The DVS experiments demonstrate that co-crystallization could significantly improve the hygroscopicity of L-CN. 

Accelerated stability of the co-crystal CUR-L-CN was conducted under 40 °C, 75% RH conditions, packaged with aluminum foil bag. After 3 months, the samples were checked via PXRD to test the physical stability, and the results are presented in Figure S3. The PXRD patterns of these samples remain unchanged, indicating that the crystalline form of the co-crystal is stable. The chemical stability was determined via high performance liquid chromatography (HPLC), and the contents of CUR in the co-crystal after stored 0, 1, 2, and 3 months were 66.4%, 66.7%, 65.9%, and 66.1%, respectively. The co-crystal can also remain chemically stable after storing under harsh condition for 3 months.

### 2.6. Powder Dissolution Studies

**Figure 8:**
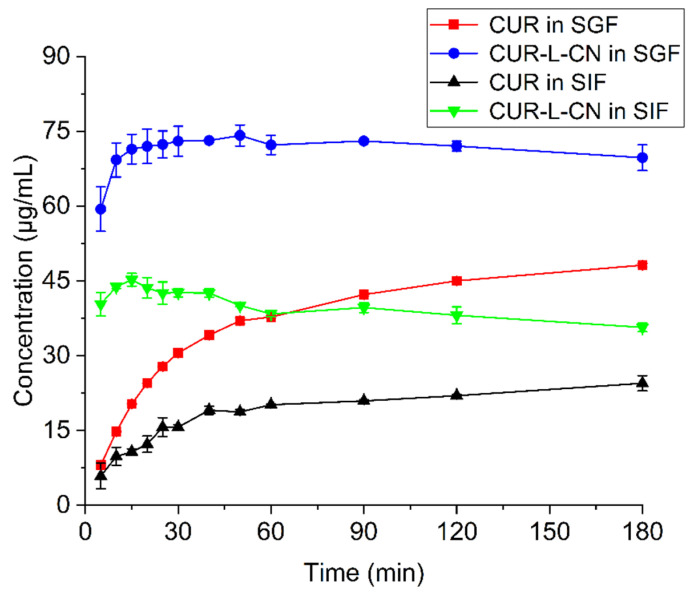
Powder dissolution profiles of CUR and CUR-L-CN at 37°. SGF = simulated gastric fluids; SIF = simulated intestinal fluids.

Powder dissolution experiments were performed to compare the water solubility of CUR and its co-crystal. The compared dissolution profiles are presented in Figure 8. CUR-L-CN presents a significantly improved dissolution rate and apparent solubility compared to that of CUR itself. CUR-L-CN dissolves quickly and reaches its maximum concentration in 20 min. In comparison, CUR dissolves much slower and the concentration continues to increase over 180 min. The maximum solubility values for CUR-L-CN and CUR are 74.2 μg/mL (CUR-L-CN in simulated gastric fluids (SGF)), 45.2 μg/mL (CUR-L-CN in simulated intestinal fluids (SIF)), 48.2 μg/mL (CUR in SGF), and 24.5 μg/mL (CUR in SIF), respectively. CUR-L-CN generates 1.5 (SGF) and 1.8 (SIF) times the maximum solubility of that of the marketed CUR crystal. In both SGF and SIF, CUR-L-CN exhibits an obvious spring–parachute effect. To understand this phenomenon, the residual powders after dissolution experiments were filtered and dried for PXRD analysis. From the PXRD plots (Figure S4), it can be seen that during the dissolution process, the co-crystal transformed into CUR, which led to a decrease in the solubility.

### 2.7. Hirshfeld Surface Analysis

**Figure 9:**
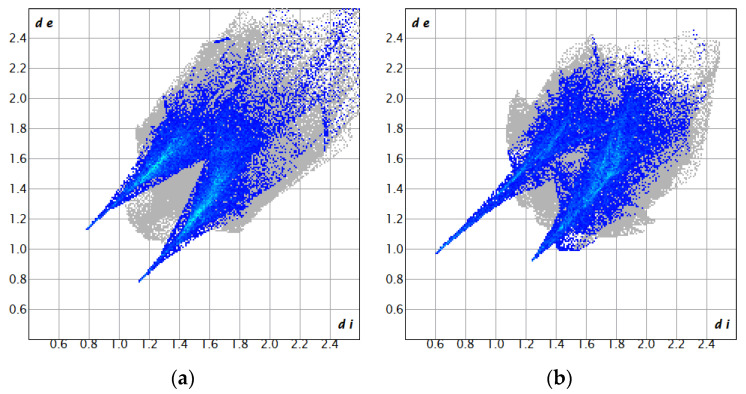
Two-dimensional fingerprints of (**a**) CUR and (**b**) CUR-L-CN.

To understand the faster dissolution rate of CUR-L-CN compared to the original CUR, we performed Hirshfeld surface analysis using Crystal Explorer 21.5 software. It generates 2D fingerprints that quantitatively represent the contribution of various types of molecular interactions during molecular stacking. Hydrophilicity is often thought to be represented by polar interactions (H···O), and the proportion of polar interactions may be used to compare the affinity for water of different materials. The 2D fingerprints of CUR and CUR-L-CN are shown in Figure 9. The distinctive sharp peaks in the CUR-L-CN profile indicate O···H/O···H interactions (26.3%). In contrast, the CUR feedstock shows fewer O···H/O···H interactions (25.5%). The formation of hydrogen bonds between CUR and L-CN enhances the ratio of polar interactions between co-crystalline molecules. Therefore, during the dissolution process, CUR-L-CN has a higher affinity for water and thus exhibits faster dissolution rates.

### 2.8. Wettability Test

**Figure 10:**
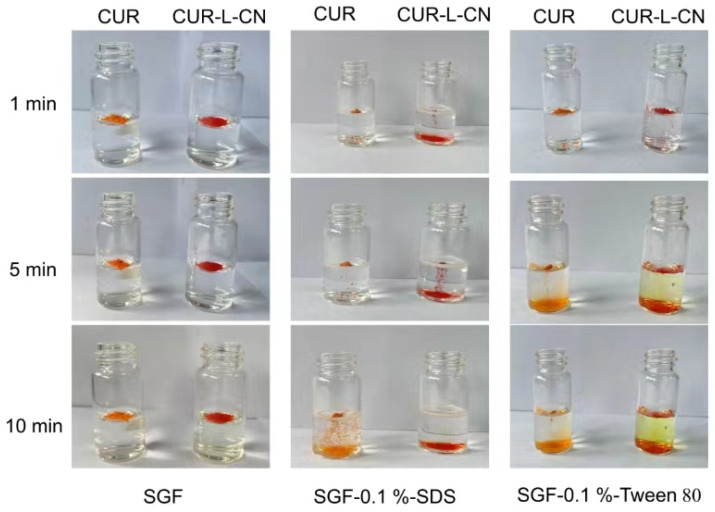
Wettability test for CUR and its co-crystal.

To confirm the hypothesis given in the section discussing Hirshfeld surface analysis, wettability tests using film flotation methods were conducted in SGF with/without surfactants. The results are presented in Figure 10. In SGF without surfactant, both CUR and CUR-L-CN could not sink into the fluid. SDS and tween 80 significantly improve the wettability of CUR and its co-crystal. In the solution of SGF containing 0.1% SDS, after 10 min, all the co-crystal samples had sunk to the bottom, while only a small quantity of CUR had sunk. In the solution of SGF containing 0.1% tween 80, CUR-L-CN also sunk much faster than that of CUR itself. Such results confirm the hypothesis (i.e., that CUR-L-CN has a higher affinity for water) derived from Hirschfeld surface analysis.

### 2.9. In Vivo Bioavailability

**Figure 11:**
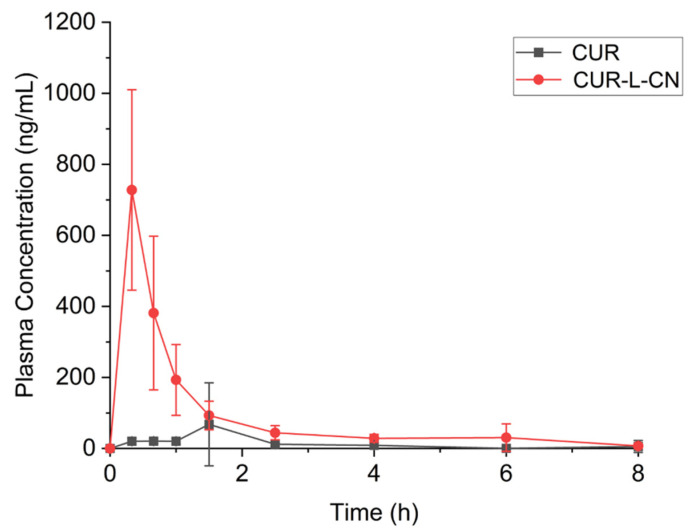
Plasma CUR concentration–time curves of CUR and CUR-L-CN.

The in vivo pharmacokinetic investigations of the CUR-L-CN co-crystal and the marketed CUR crystal were carried out in fasted male rats. In vivo, CUR is easily conjugated, and it can be conjugated with glucuronic acid and sulfate to form monoglucuronides, monosulfates, and mixed glucuronide/sulfates. Hence, the plasma samples were handled with β-glucuronidase/sulfatase before testing. Pharmacokinetic parameters and plasma concentration–time curves of CUR and CUR-L-CN are shown in Table 2 and Figure 11. The C_max_ (plasma peak concentration) of the co-crystal and the marketed CUR are 727.8 ng·mL^−1^ and 67.9 ng·mL^−1^, respectively. The CUR-L-CN co-crystal generates 10.7 times more C_max_ than that of CUR itself. In addition, the AUC_0−8h_ (area under the concentration curve) values of CUR-L-CN and CUR are 692.5 ng·h·mL^−1^ and 109.9 ng·h·mL^−1^, respectively. The co-crystal generates 6.3 times more AUC_0−8h_ than that of CUR itself. The PK studies demonstrate that the co-crystal significantly improves the oral absorption of CUR, originating from the much faster dissolution rate.

## 3. Discussion

Several co-crystals of CUR have been reported, but none of them presented markedly improved absorption. There are three main considerations in the selection of the co-crystal ligands in this work. First, it must be able to form a co-crystal with CUR. In this sphere, ligand selection is due to the supramolecular synthon of CUR, and the hydrogen bonding between the phenolic hydroxyl group and the carboxylic acid negative ion (O–H···O^−^) is one of the most common synthons in the co-crystal design. Second, the chosen ligands must be safe such that they could be wildly used in human nutrition. Finally, it would be better if the ligand and CUR had a synergistic effect. Finally, we found that L-CN was the suitable ligand and the co-crystal was successfully prepared. 

**Figure 12:**
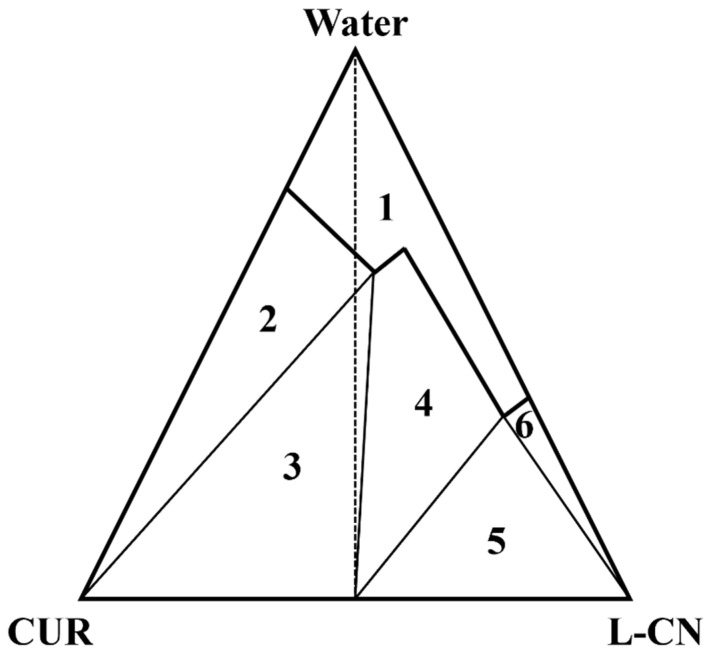
Ternary phase diagrams for systems comprising CUR, L-CN, and water [43].

High hygroscopicity is one of the application difficulties of L-CN. The CUR-L-CN co-crystal significantly reduced the hygroscopicity of L-CN. However, when the relative humidity of the environment was higher than 75%, the CUR-L-CN co-crystal would be dissociated. This dissociation may be caused by the difference in water solubility between CUR and CN. This phenomenon can be explained by the ternary phase diagram of API, ligand, and water. William Jones et al. [43] provides a ternary phase diagram model to explain the mechanism of co-crystal dissociation at high humidity. The hypothetical ternary phase diagram of CUR, L-CN, and water referred to in the reported paper is presented in Figure 12. In region 1, CUR and L-CN are dissolved in water. The other regions contain solid phases at equilibrium: 2, pure CUR; 3, CUR + CUR-L-CN; 4, pure CUR-L-CN; 5, CUR-L-CN + L-CN; 6, pure L-CN. From the ternary phase diagram, it can be concluded that as the water content in the system increases, the CUR-L-CN co-crystal gradually dissociates, forming a mixture of CUR-L-CN and CUR.

The results of the dissolution experiments demonstrate that the CUR-L-CN co-crystal has a much faster dissolution rate and higher apparent solubility than CUR. The differences in solubility are observed to be attributable to different pH values. For both CUR and its co-crystal, their solubilities in SGF are higher than that in SIF. Hanne Hjorth Tonnesen and Jan Karlsen. [44] reported the aqueous stability of CUR in different pH solutions. CUR presented much better aqueous stability in a low-pH solution than in a high-pH solution. The pH value of SIF is much greater than that of SGF. Hence, CUR is more stable in SGF than in SIF. Hence, the solubility difference may result from the differences in the stability of CUR at different pH conditions.

The PK experiments of CUR and CUR-L-CN were performed on rats, and CUR-L-CN exhibited significantly improved bioavailability compared to that of CUR. CUR has a much longer T_max_ than that of the co-crystal. The T_max_ of CUR and CUR-L-CN are 90 and 20 min, respectively. Such results indicate that CUR is absorbed mainly in the intestine, and CUR-L-CN is absorbed in the stomach. The dissolution rate may result in a difference in the absorption site. It is well known that the intestinal absorption of CUR is extremely low [45]. Gastric absorption may be one of the reasons for the increased bioavailability of CUR-L-CN. 

## 4. Materials and Methods

### 4.1. Materials

CUR with a purity over 98% was purchased from Innochem (Beijing, China) Co., Ltd. L-CN (purity 98%) was supplied by Aladdin (Shanghai, China) Co., Ltd. All solvents used in this work were analytic reagent grade and they were purchased from Sinopharm Chemical Reagent (Shanghai, China) Co., Ltd.

### 4.2. Preparation of CUR-L-CN Co-Crystal

CUR-L-CN co-crystal was prepared via liquid-assisted grinding. A total of 36.8 mg CUR, 16.1 mg L-CN, and 10 μL ethanol were mixed and mechanically milled via a high-energy ball mill instrument (Tissuelyser-II, Jingxin Co., Ltd., Shanghai, China). Finally, the milled wet samples were dried in vacuum at room temperature for 12 h to obtain the co-crystal.

### 4.3. Preparation of Single Crystal

CUR-L-CN single crystal was obtained via slow evaporation. Excess CUR-L-CN powder was added in 1 mL of a solution of ethanol and ethyl acetate (*v*/*v*, 3:7). Then, the remaining solids were removed, and the supernatant slowly evaporated at room temperature. After three days, red block-shaped crystals were obtained (Figure S5). 

### 4.4. PXRD

A D8 advanced (Bruker Co., Ltd., Saarbrucken, Germany) X-ray diffractometer was used to collect the PXRD profiles. The instrument was equipped with Cu Kα radiation, and the voltage and current were set to 40 kV and 40 mA, respectively. Samples were checked at 20 °C with a scan rate of 0.05 s/step. The detection range is set to 3–40° 2θ. RINT Rapid was used to image and integrate the obtained data. MDI Jade 6.0 was used as a peak analysis software.

### 4.5. SCXRD

A Bruker Apex II CCD diffractometer was applied to determine the crystal structure of CUR-L-CN co-crystal. The experiment was performed at 170 K via a Mo-Kα radiation (λ = 0.71073 Å). SAINT integrates and scales the collected data. The absorption effects were corrected by SADABS. Olex2 1.5 software was provided for solving and refining the structure. SHELX-2017 software was utilized to refine the structure via the full matrix least squares technique. Isotropic atomic displacement parameters are applied in the refining process of the non-hydrogen atoms. The cycling model is applied in the refining process of the hydrogen atoms, and the positions of the hydrogen atoms are determined by calculation. The co-crystal graphs were created using Mercury 2023 1.0 software [46] from CCDC. The cif document of the CUR-L-CN co-crystal has been recorded in the CCDC and its CCDC number is 2333746.

### 4.6. DSC

DSC Q2000 equipment (TA Instruments, New Castle, DE, USA) was used to collect the DSC data. Nitrogen gas was applied, and its flow rate was set to 50 mL/min. A 3–5 mg sample was put in an aluminum pan; then, the pan was sealed and heated at a rate of 10 °C/min. Before the DSC experiments, indium and tin were used as the standards to calibrate the instrument via the two-point calibration method. The TA Universal Analysis 4.5 A software was used to analyze the data. 

### 4.7. TGA

A TGA 55 instrument (TA Instruments, New Castle, DE, USA) was utilized to obtain the TGA data. A 3–5 mg powder sample was put in an alumina oxide pan without cover; then, the pan was heated from 25 °C to 410 °C at a heating rate of 10 °C/min. Nitrogen gas was applied as purge gas, and its flow rate was set as 20 mL/min. TA TRIOS 5.1 software was applied to process and analyze the collected TGA data.

### 4.8. FTIR Spectroscopy

A Nicolet FTIR 750 spectrometer was used to conduct the FTIR experiments. The FTIR data were collected by a diamond module in a range of 4000 to 400 cm^−1^. The average scan numbers of each sample were 32 and the spectral resolution was 4 cm^−1^. 

### 4.9. DVS

The DVS profiles of different solid forms were tested on a SMS Intrinsic instrument, UK. To make sure each sample particle size was uniform, all samples were screened at 100 mesh (ΦA = 0.148 mm). Samples of 10–20 mg were used in the experiments. The DVS data were collected in an RH range of 0–95%. If the sample mass changes by less than 0.02% in 60 min, the next RH is conducted in a 5% step.

### 4.10. Stability Test

The physical and chemical stability of CUR-L-CN were studied under 40 °C, 75% RH. After 0, 1, 2, and 3 months, the samples were taken out and checked via PXRD and HPLC. 

### 4.11. Powder Dissolution Studies

The solubility and dissolution rate of CUR and its co-crystal were compared via powder dissolution experiments. A mini bath dissolution device was used in the experiments, and a Julabo-5 heater was equipped for heating. The experiments were conducted at 37 °C with a rotational speed of 50 rpm. To minimize the effect of variations in particle size and morphology, the marketed CUR crystal and CUR-L-CN co-crystal were screened at 100-mesh. The dissolution rate experiments were measured in SGF and SIF containing 0.5% tween 80. SGF was prepared by diluting 16.4 mL of 10% HCl solution to 1000 mL with pure water. To prepare SIF, 6.8 g of KH_2_PO4 was dissolved in 500 mL of water; then, the pH was adjusted to 6.8 with NaOH solution with a concentration of 0.1 mol/L. At specified time points, i.e., 5 min, 10 min, 15 min, 20 min, 25 min, 30 min, 40 min, 50 min, 60 min, 90 min, 120 min, and 180 min, 0.5 mL of solution was withdrawn with a syringe. The collected solution was filtered through a 0.45 µm nylon filter and then subjected to HPLC determination. The dissolution studies of each solid form were conducted in triplicate (*n* = 3). After dissolution experiments, the residual samples were filtered and dried under vacuum for PXRD analysis. 

### 4.12. Wettability Test

The wettability test was performed in pure SGF and SGF with 0.1% sodium dodecyl sulfonate (SDS) or tween 80 using film flotation methods. A total of 10 mg CUR and its co-crystal were put on the solution, and the wettability was compared by calculating the powder sinking speed. 

### 4.13. HPLC Analysis

An Agilent 1260 series HPLC (Agilent Technologies Co., Ltd., Santa Clara, CA, USA) was applied to determine the concentration of CUR in the stability and dissolution experiments. The equipment has a diode array detector. An Agilent Zorbax Eclipse Plus C18 column was used in the experiments; it has a diameter of 4.6 mm, a length of 150 mm, and a filling aperture of 5 µm. The mobile phase contained 52% glacial acetic acid (4%) aqueous solution and 48% acetonitrile. The column temperature was set as 35 °C, and the UV-vis wavelength was set to 430 nm. A 10 µL sample was injected each time and the flow rate of the mobile phase was aet as 1 mL/min. 

### 4.14. Pharmacokinetic Study

Twelve Sprague Dawley male rats weighted at 200 ± 50 g were given CUR-L-CN co-crystal and pure CUR (six rats for each formulation) orally with a dose of 200 mg/kg. The samples were dispersed in soybean oil (600 mg CUR in 10 mL of oil), and each rat was given about 1 mL of the suspension by gavage. After administration, approximately 0.4 mL of blood was collected from the orbit of the rats into heparinized tubes at 20 min, 40 min, 1 h, 1.5 h, 2.5 h, 4 h, 6 h, and 8 h. Plasma samples (100 μL) were precisely aspirated and vortexed with 50 μL of β-glucuronidase and sulfatase buffer at 37 °C for 60 min. Plasma samples were protein precipitated by adding 0.45 mL of methanol (0.2% acetic acid). After 10 min of shaking, the samples were centrifuged at 14,000 rpm for 3 min. Subsequently, the supernatants were analyzed via HPLC/MS. All experimental procedures were approved by the Animal Ethics Committee. The oral absorption of CUR and CUR-L-CN co-crystal was compared by calculating their AUC and C_max_.

### 4.15. Bioanalytical Method

The CUR plasma concentration was determined by A Sciex Triple Quad 4500 LC-MS equipment. HPLC methods and conditions were the same as 4.13. A 10 µL sample was injected each time, and the flow rate of the mobile phase was set as 0.6 mL/min. The multiple reaction monitoring mode was utilized to conduct the detection and quantification, with *m*/*z* 369.2 → 177.1 for CUR.

### 4.16. Data Analysis

The AUC_0–t_ was calculated from plasma concentrations observed from 0 to 8 h using a linear trapezoidal approach. 

## 5. Conclusions

CUR is a natural polyphenolic compound with various pharmacological activities, and liver protection is one of the most famous and important effects. Low water solubility and bioavailability limit its clinical application. To improve the bioavailability of CUR, we successfully prepared a new co-crystal of CUR and L-CN. The co-crystal was fully characterized via PXRD, FTIR, DSC, TGA, and DVS. The crystal structure was analyzed by SCXRD. Dissolution experiments were conducted in SGF and SIF. CUR-L-CN exhibited significantly faster dissolution rates than that of pure CUR. Hirshfeld surface analysis and a wettability test were discussed to understand the faster dissolution rate of CUR-L-CN. The formation of hydrogen bonds between CUR and L-CN enhances the ratio of polar interactions among the co-crystalline molecules, resulting in a higher affinity for water and faster dissolution rates of CUR-L-CN. Finally, pharmacokinetic studies of CUR-L-CN and CUR itself were performed on rats. The results showed that compared to pure CUR, CUR-L-CN exhibited 6.3-times-higher AUC_0–8h_ and 10.7-times-higher C_max_. Considering that both CUR and L-CN have hepatoprotective effects, and that the co-crystal also greatly improves the absorption of CUR and reduces the hygroscopic properties of L-CN, the CUR-L-CN co-crystal has great potential in hepatoprotective applications. 

## Figures and Tables

**Table 1:** Crystallographic data and details of refinement for the CUR-L-CN co-crystal.

Name	CUR-L-CN
Formula	C_56_H_70_N_2_O_18_
Formula weight (Da)	1059.14
T (K) *	170
Crystal system	Monoclinic
Space group	P2_1_
a (Å)	17.1370(11)
b(Å)	5.6406(4)
c/Å	27.393(2)
α (°)	90
β (°)	101.063(2)
γ (°)	90
V (Å^3^)	2598.7(3)
Z	2
D_cal_ (g/cm^3^)	1.354
R_int_	0
λ (Mo-Kα, Å)	0.71073
Independent reflns	10414
GOOF	1.045
R_1_	0.1051
wR_2_	0.1587
CCDC number	2,333,746

* Acronyms: T = temperature; V = volume; D = density; GOOF = goodness of fit, CCDC = Cambridge crystallographic data center.

**Table 2:** Pharmacokinetic parameters of CUR and CUR-L-CN.

	CUR	CUR-L-CN
T_max_ (min) *	90	20
C_max_ (ng·mL^−1^)	67.9	727.8
AUC_0–8_ (ng·h·mL^−1^)	109.9	692.5 **

* T_max_ = time to peak plasma concentration; C_max_ = peak plasma concentration; AUC = area under the concentration curve. ** indicates significantly different between CUR-L-CN and CUR at the 0.01 level.

## Data Availability

Data is contained within the article and Supplementary Material.

## Supplementary Materials

The following supporting information can be downloaded at: https://www.mdpi.com/article/10.3390/ph17040489/s1, Figure S1: TGA plots of CUR, L-CN, and CUR-L-CN; Figure S2: PXRD patterns of CUR-L-CN after DVS experiment; Figure S3: PXRD patterns of CUR-L-CN after stored under 40 °C, 75%RH condition for 0, 1, 2, and 3 months; Figure S4: PXRD patterns of residual powders after dissolution experiments. Figure S5: Single crystal picture of CUR.
