# Supplementary material for: Improving the Dissolution Rate and Bioavailability of Curcumin via Co-Crystallization
Source: Pharmaceuticals (Basel). 2024 Apr 11;17(4):489. doi: 10.3390/ph17040489 (PMC11053631; doi:10.3390/ph17040489)
Supplement: Supplementary file 1 [file pharmaceuticals-17-00489-s001.zip › pharmaceuticals-2928796-supplementary.pdf]

Supplementary material:

Improving the dissolution rate and bioavailability of curcumin via Co-crystallization

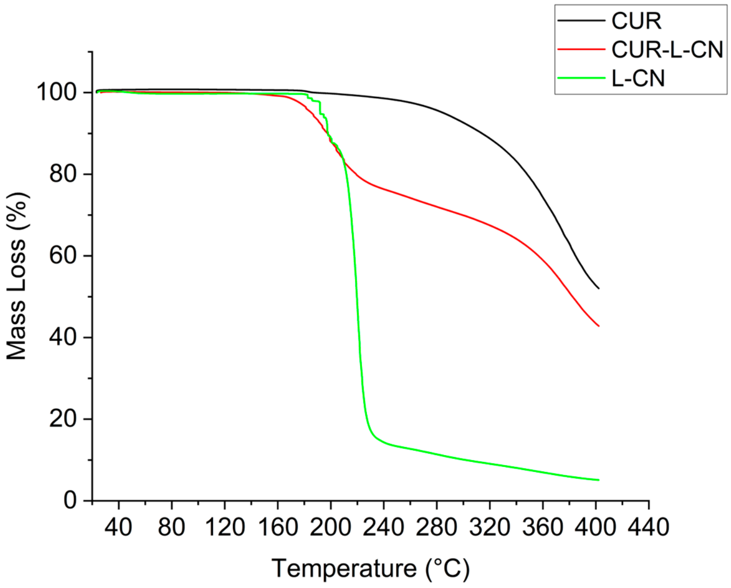

Figure S1. TGA plots of CUR, L-CN, and CUR-L-CN.

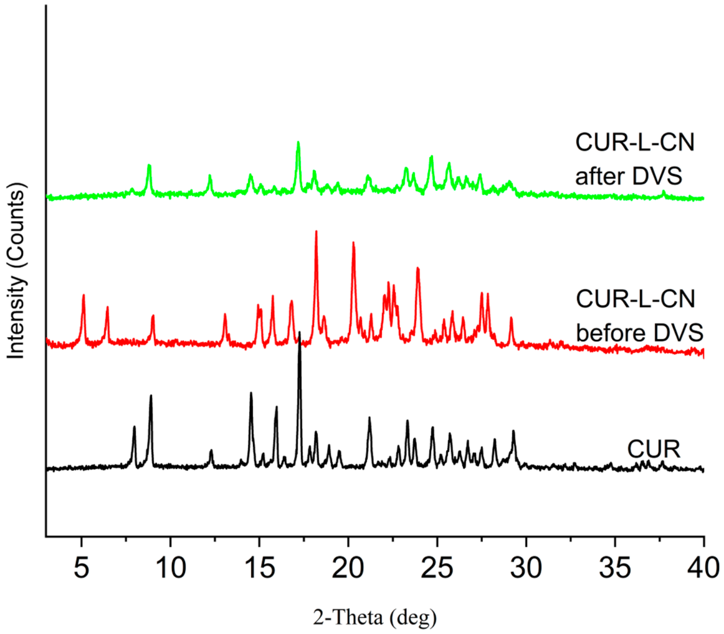

Figure S2. PXRD patterns of CUR-L-CN. after DVS experiment.

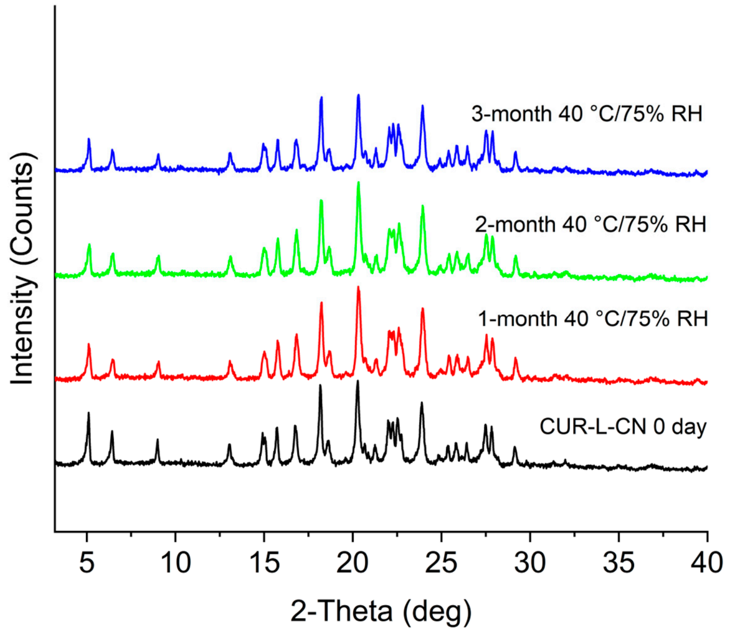

Figure S3. PXRD patterns of CUR-L-CN after stored under 40°C, 75%RH condition for 0, 1, 2, and 3 months.

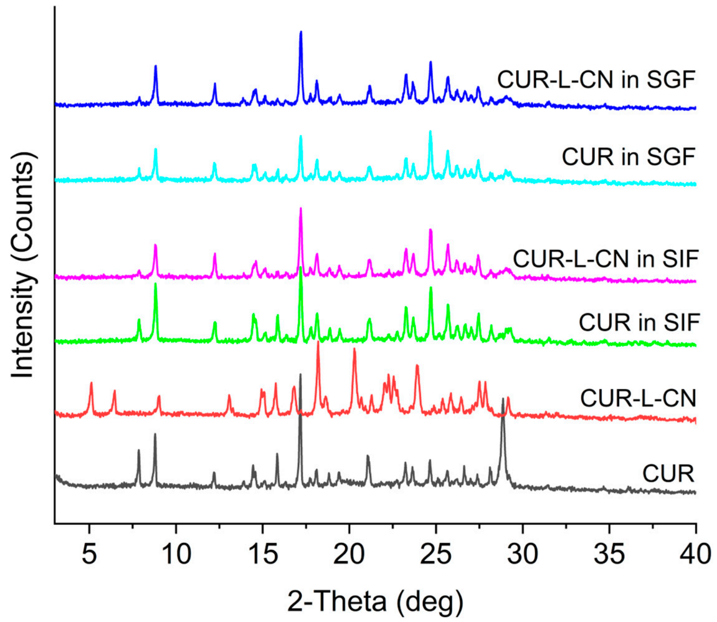

Figure S4. PXRD patterns of residual powders after dissolution experiments.

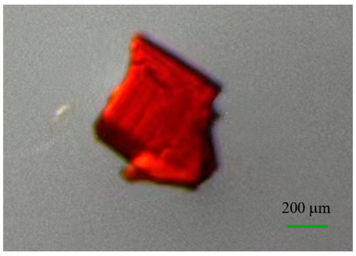

Figure S5. Single crystal of CUR.
